# Spin cascade and doming in ferric hemes: Femtosecond X-ray absorption and X-ray emission studies

**DOI:** 10.1073/pnas.2009490117

**Published:** 2020-08-26

**Authors:** Camila Bacellar, Dominik Kinschel, Giulia F. Mancini, Rebecca A. Ingle, Jérémy Rouxel, Oliviero Cannelli, Claudio Cirelli, Gregor Knopp, Jakub Szlachetko, Frederico A. Lima, Samuel Menzi, Georgios Pamfilidis, Katharina Kubicek, Dmitry Khakhulin, Wojciech Gawelda, Angel Rodriguez-Fernandez, Mykola Biednov, Christian Bressler, Christopher A. Arrell, Philip J. M. Johnson, Christopher J. Milne, Majed Chergui

**Affiliations:** ^a^Laboratoire de Spectroscopie Ultrarapide, Institut des Sciences et Ingéniéries Chimiques and Lausanne Centre for Ultrafast Science, Ecole Polytechnique Fédérale de Lausanne, 1015 Lausanne, Switzerland;; ^b^Swiss Free Electron Laser, Paul-Scherrer-Institut (PSI), 5232 Villigen PSI, Switzerland;; ^c^Institute of Nuclear Physics, Polish Academy of Sciences, 31-342 Kraków, Poland;; ^d^European X-ray Free Electron Laser, D-22869 Schenefeld, Germany;; ^e^Faculty of Physics, Adam Mickiewicz University, 61-614 Poznan, Poland

**Keywords:** ultrafast, X-ray spectroscopy, ferric hemoproteins, spin states, doming

## Abstract

The structure–function relationship is at the heart of biology, and major protein deformations are correlated to specific functions. For ferrous heme proteins, doming is associated with the respiratory function in hemoglobin and myoglobins. Cytochrome *c* (Cyt c) has evolved to become an important electron-transfer protein in humans. In its ferrous form, it undergoes ligand release and doming upon photoexcitation, but its ferric form does not release the distal ligand, while the return to the ground state has been attributed to thermal relaxation. Here, by combining femtosecond Fe K_α_ and K_β_ X-ray emission spectroscopy (XES) with Fe K-edge X-ray absorption near-edge structure (XANES), we demonstrate that the photocycle of ferric Cyt c is entirely due to a cascade among excited spin states of the iron ion, causing the ferric heme to undergo doming, which we identify. We also argue that this pattern is common to a wide diversity of ferric heme proteins, raising the question of the biological relevance of doming in such proteins.

## Significance

The structure–function relationship in central to biology, while the structural dynamics are driven by electronic changes. Doming of ferrous heme proteins, which is central to the respiratory function of hemoglobin, ensues from populating high-spin states. However, for ferric heme proteins, doming was excluded. Here, we show that high-spin states are populated in photoexcited ferric cytochrome *c*, and we present evidence for doming. We also conclude that photo- or thermally activated doming occurs in a wide variety of ferric heme proteins, calling for a deeper understanding of its role in their respective functions.

**Fig. 1.**
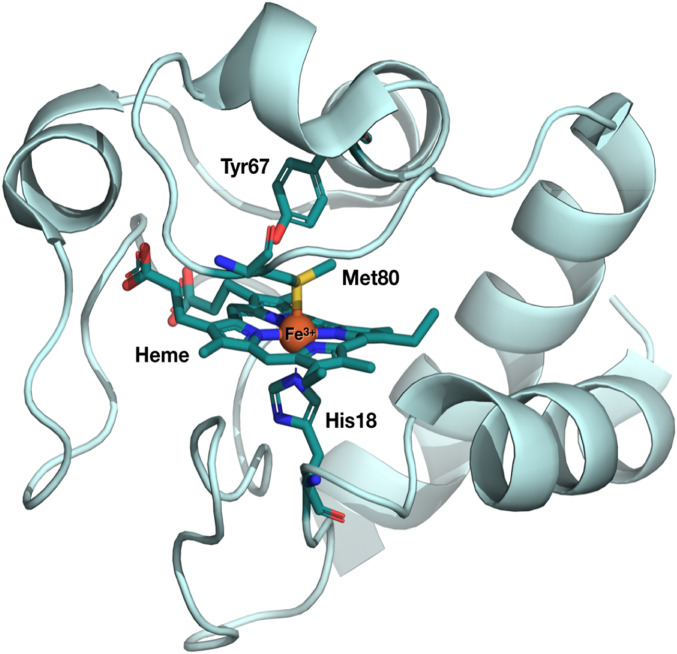
Crystal structure of ferric Cyt c. The heme and the Met80, His18, and Tyr67 amino acid residues ligated to the Fe heme are highlighted as sticks (Fe [orange], C [teal], N [blue], O [red], S [yellow]). The structure was obtained from the Protein Data Bank, under ID code 1HRC (18). The Fe atom at the center of the porphyrin is coordinated by four pyrrole (N_p_) atoms and by the distal Met80 and the proximal His18 ligands.

Cytochrome *c* (Cyt c) is a small protein that mediates the electron transfer (ET) from Cyt c reductase to Cyt c oxidase (1). It also plays a role in apoptosis and its conformationally dependent peroxidase activity (2–4). Cyt c consists of an iron protoporphyrin IX complex, with the central Fe atom hexacoordinated by the four N pyrrole atoms (Np) of the porphyrin, a distal methionine ligand (Met80) and a proximal histidine (His18) ligand (Fig. 1). The latter anchors the heme to the protein peptide chain and is also linked to the protein via two thioether covalent bonds with Cys14 and -17. Several studies have been conducted aimed at relating the ET properties of Cyt c to structural factors that govern, e.g., the reduction potentials and the electronic coupling between donor and acceptor (5–7). The Cyt c heme is characterized by a large ruffling distortion (an out-of-plane [OOP] distortion of the porphyrin; *SI Appendix*, Fig. S1*B*) induced by the protein fold and the heme motif. Heme ruffling is the dominant OOP deformation in *c*-type cytochromes involved in ET (8–10) and in nitrophorins involved in NO transport (11–13). These OOP distortions are energetically unfavorable (14), but as they have been conserved through evolution, it was proposed that ruffling is crucial for tuning the reduction potential of the protein and therefore its ET properties (15–17). NMR studies (15) showed that ruffling destabilizes all three occupied Fe 3d-based molecular orbitals (*SI Appendix*, Fig. S1*B*), and it was hypothesized that this controls the redox potential.

Another major heme deformation that has been conserved through evolution is doming (*SI Appendix*, Fig. S1*C*). It has been well characterized and correlated to functions such as oxygen storage and transport in hemoglobins (Hbs) and myoglobins (Mbs) (19, 20). Doming results from the occupation of antibonding Fe d-orbitals, i.e., formation of higher metal spin states (*SI Appendix*, Fig. S1*C*). In the case of ferrous hemoproteins (Hb, Mb, or Cyt c), upon distal ligand release from the Fe atom, the pentacoordinated heme adopts a domed deoxyMb configuration in a high-spin (HS) quintet state (*SI Appendix*, Fig. S1*C*). This and the reverse process (from domed to planar) are considered the initial molecular-level events of the respiratory function in Hb (19).

Ever since it was discovered that ligand detachment from ferrous hemes can be induced with high quantum yield by absorption of ultraviolet (UV)-visible light (21), photoexcitation has been used to mimic the basic events of the respiratory function, i.e., the ligand detachment from, and recombination to the Fe atom. This was particularly the case with the advent of ultrafast spectroscopy as ferrous heme proteins were among the first systems ever to be investigated by such a method (22). By photoexciting the heme porphyrin either into its Q-band (visible) or Soret-band (UV), or even into higher lying states, the distal ligand detachment–recombination events in ferrous hemes have been monitored using various optical (UV, visible, infrared [IR], Raman) probes (23–26). It was concluded that prompt photodissociation of the ligand from the low-spin (LS) planar heme occurs, accompanied by a simultaneous doming of the heme porphyrin, i.e., formation of the HS quintet deoxyMb. Evidence of ligand dissociation and doming are based on three types of observables: 1) the transient UV-visible absorption (TA) spectra in the region of the Q- and Soret-bands reproduce the difference between the steady-state UV-visible absorption spectra of the HS pentacoordinated domed deoxyMb and the LS hexacoordinated ferrous planar ligated hemes (27); 2) the transient resonance Raman studies, which exhibit the shift of the Fe–His bond frequency from its value in the planar ligated heme (∼265 cm^−1^) to its value in the deoxyMb form (∼220 cm^−1^) (24, 28); and 3) ligand dissociation is also witnessed by the appearance of the IR bands of the unbound diatomic ligand in carboxymyoglobin (MbCO) (29–31), nitrosylmyoglobin (MbNO) (32), and oxymyoglobin (MbO_2_) (30, 33).

In the case of ferric heme proteins such as metmyoglobin (metMb) (27), Cyt c (24), cyanomyoglobin (MbCN) (34), azidomyoglobin (MbN_3_) (35), and the protein sensor oxy-FixLH (36), either one or more of the above observables for heme doming and/or ligand release were missing, leading to the conclusion that ligand dissociation and doming do not take place in ferric hemes. Specifically for the much-studied ferric Cyt c, the description prevailed that the electronically photoexcited heme decays to high-lying vibrational levels of the ground state, which then undergoes thermal relaxation (7, 24, 26, 37, 38). It was further concluded (25) that the latter causes heme photoreduction (6, 25, 28), but the origin of the reducing electron is unclear, although nearby amino acid residues, such as Tyr67 (Fig. 1), have been proposed as possible candidates (5).

The scenario of a pure thermal relaxation in ferric hemes was recently challenged by ultrafast IR to UV-visible TA studies on ferric MbCN (34), metMb (39), and MbN_3_ (35), suggesting that a cascade through excited spin states may be involved, although not ruling out a partial parallel thermal relaxation pathway. These states lie between the lowest unoccupied molecular orbital (LUMO), which corresponds to the Q-state, and highest unoccupied molecular orbital (HOMO), which corresponds to the ground state of the heme porphyrin, and are due to population of metal d-orbitals (*SI Appendix*, Fig. S3) (40). They cannot be optically accessed because of selection rules. In fact, UV-visible probes only monitor the HOMO–LUMO (e.g., the Q- and Soret-bands) of the porphyrin ring, and they are not sensitive to spin states of the metal. Therefore, the above conclusion that relaxation of the photoexcited heme proceeds via excited spin states (34, 35, 39) was not based on their direct observation but was inferred from the relaxation kinetics and, whenever this was the case, on IR spectral features that show up upon photoexcitation (34, 35).

X-ray absorption (XAS) and emission spectroscopy (XES) can circumvent the limitations of optical spectroscopies as they are element-selective and structure- and spin-sensitive (41, 42). In recent years, time-domain (femtoseconds to picoseconds) X-ray spectroscopies have emerged as powerful tools to probe the electronic structure changes and the structural dynamics of (bio)molecules and of materials with the additional advantage that they can detect optically silent transient states (43). Quasi–steady-state (44–46) and femtosecond-to-picosecond Fe K-edge X-ray near edge structure (XANES) have already been used to probe the photoinduced electronic and structural changes in photoexcited ferrous hemoproteins, such as MbCO (47, 48), MbNO (49), and Cyt c (50). Similar studies were also performed at the Co K-edge on cyanocobalamin (vitamin B_12_) using polarized XAS (51, 52). All of these studies concluded that ligand dissociation and formation of a domed porphyrin take place. Furthermore, XES has emerged as a reliable marker of the spin state of the transition metal atom (number of unpaired 3d electrons) via the K_β_ lines (3p→1s emission) that reflect the 3p–3d exchange interactions and exhibit an intensity decrease and blue energy shift of the K_β_ line with increasing spin, while the K_β’_ sideband increases in intensity, as can be seen in the spectra of reference compounds in *SI Appendix*, Fig. S2 *B* and *C* (41). The 2p–3d interactions are weaker than the 3p–3d ones, and yet K_α_ XES is also a marker of spin via the linear dependence of the K_α1_ line full-width at half-maximum (FWHM) and the exchange interaction of the core hole with the number of unpaired spins in the valence shell, up to S = 3/2 (53). Femtosecond Fe K_β_ XES studies revealed the details of the ultrafast spin cross-over (SCO) in photoexcited [Fe(bpy)_3_]^2+^ (54) and was also used to identify the HS (S = 2) deoxy heme product of photoexcited ferrous Cyt c after dissociation of its distal methionine ligand (50). In the present work, we specifically address the relaxation cascade in photoexcited ferric horse heart mitochondrial Cyt c using element-specific and structure- and spin-sensitive methods such as femtosecond Fe K_α_ and K_β_ XES (*SI Appendix*, Fig. S2*A*) and femtosecond Fe K-edge XANES at the Swiss Free Electron Laser (SwissFEL, Villigen, Switzerland) and the European XFEL (Eu-XFEL, Hamburg, Germany). We show that the decay of the photoexcited heme in ferric Cyt c entirely proceeds via HS states of the metal, which we identify by both their electronic XES and structural XANES signatures. Details of the experimental procedures are given in *SI Appendix*.

## Results and Discussion

**Fig. 2.**
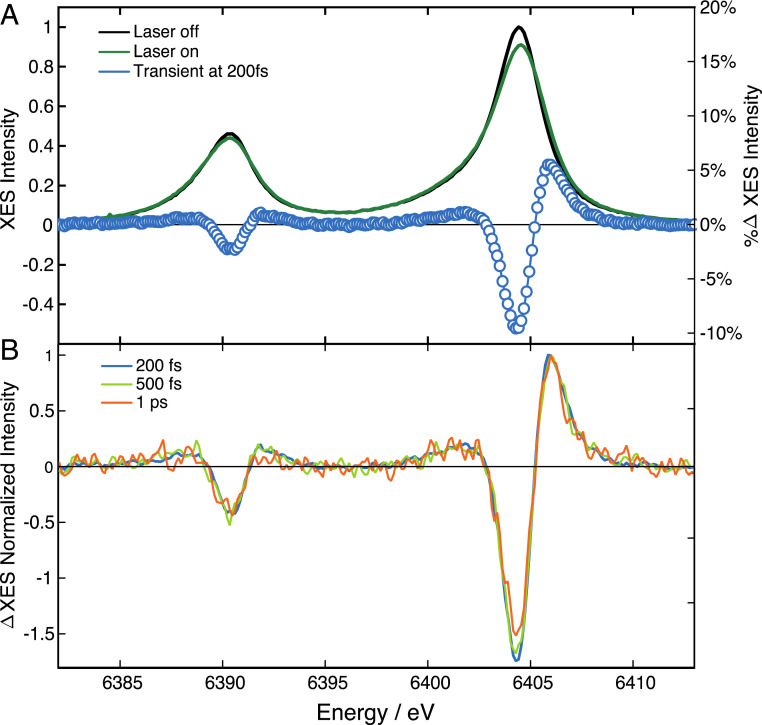
(*A*) Steady-state (laser-off) Fe-K_α_ emission spectrum of ferric Cyt c in room temperature aqueous solutions at pH 7, showing the K_α1_ (6,404 eV) and K_α2_ (6,391 eV) lines (black trace). Shown are the laser-on spectrum at 200-fs time delay (green trace) and the transient XES (laser-on minus laser-off) spectrum at 200-fs time delay after excitation at 400 nm (blue dots). (*B*) Transient (laser-on minus laser-off) spectrum at 200 fs (blue), 500 fs (light green), and 1 ps (orange), normalized to their maximum intensity.

Ferric Cyt c has a LS (S = 1/2) ground state with five electrons in the Fe-d_xy_, d_xz_, d_yz_ (t_2g_) orbitals (*SI Appendix*, Fig. S3). The intermediate spin (IS) (S = 3/2) and HS (S = 5/2) can be formed when one and two electrons, respectively, occupy the d_z2_ and d_x2-y2_ antibonding orbitals. This leads to an elongation of the Fe–N_p_ bonds and therefore doming. The latter is expected to be larger for S = 5/2 (two electrons in the antibonding orbitals) than for S = 3/2 (only one electron in the antibonding orbitals) (55). The electronic signature of the IS/HS state is provided by the transient K_α_ and K_β_ XES spectra. K_α_ XES is one order of magnitude more intense than the K_β_ emission and further away from the elastic peak, suffering less from the inelastic background signal. Laser-off (*t* < 0) and laser-on (*t* = 200 fs) K_α_ spectra are shown in Fig. 2*A*, along with the corresponding transient (blue circles). Transients at later time delays are shown in Fig. 2*B* and a more complete set of laser-on spectra and their transients is given in *SI Appendix*, Fig. S4, and *SI Appendix*, Fig. S5, *Upper* and *Lower Left*, which show zooms into the evolution of the laser-on K_α1_ line. It can be seen that its broadening is largest at the earliest time delays, and it decreases thereafter. In addition, it is more pronounced on the blue wing than on the red, as expected for a spin increase (41, 53). In the absence of reference K_α_ spectra of ferric heme LS, IS, and HS states and in order to quantify the broadening, we fitted the laser-off and laser-on spectra using an asymmetric pseudo-Voigt line-shape (described in *SI Appendix*, section 6) (56), which best reproduces the emission profile (*SI Appendix*, Fig. S5, *Top*). The results of the fit (*SI Appendix*, Fig. S5, *Upper Right* and *Lower Right*) are shown separately from the actual experimental line-shapes (*SI Appendix*, Fig. S5, *Upper Left* and *Lower Left*) for the sake of clarity. However, as an example, *SI Appendix*, Fig. S6 shows the result of the fit of the K_α_ transient at 200 fs: the difference of the fitted laser-off and laser-on K_α1_ lines is compared with the actual transient showing very good agreement above 6,403 eV. In fitting the laser-on K_α1_ line, a broadening of ∼0.7 eV was retrieved, which is of the order of magnitude expected for a transition from a LS to a HS state (41, 53). Since it was reported that the K_α_ line-shape does not significantly change with spin beyond S = 3/2 (53), we can only conclude that the final state has S ≥ 3/2.

**Fig. 3.**
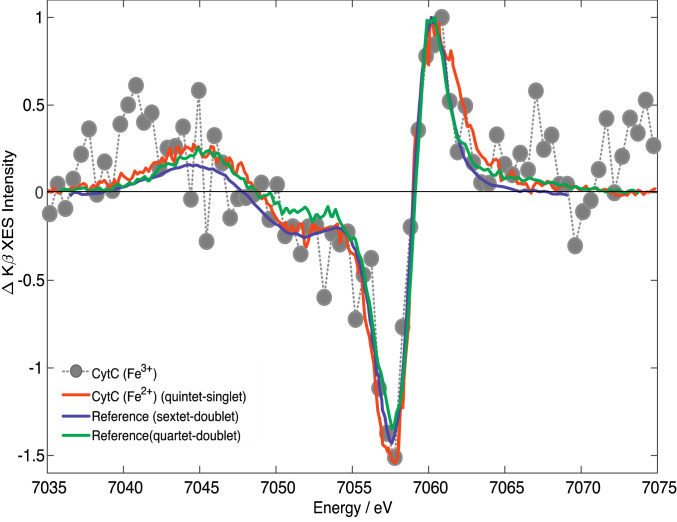
Normalized transient K_β_ XES of ferric Cyt c recorded at 200 fs (gray dots) compared with the steady-state difference K_β_ XES spectra of sextet [Fe(III)(TPP)Cl] minus doublet [Fe(III)(bpy)_3_] (purple) and quartet [Fe(III)(PTC)Cl] minus doublet [Fe(III)(bpy)_3_] (green) from refs. 54, 58 (PTC, phthalocyanine chloride; TPP, tetraphenylporphyrin). We also compare our results with the digitized transient spectrum of ferrous Cyt c at 600-fs time delay (red) from ref. 50. Note that the latter and the steady-state difference spectra were red-shifted by ∼1 to 2 eV to match the present data points.

K_β_ XES is better established as a marker of spin states because of the stronger 3p–3d interaction (*SI Appendix*, Fig. S2), but it suffers from a poor signal-to-noise ratio as it rides on a high background of elastically scattered light (57). Fig. 3 shows the K_β_ transient at 200-fs time delay. Despite the noise, its shape is clearly reminiscent of the signature of the HS (S = 2) state reported for [Fe(bpy)_3_]^2+^ (54) and ferrous Cyt c (50), whose transient at 600 fs is also shown (Fig. 3, red trace). In comparing the transients, the ferrous ones were shifted by 1 to 2 eV to the red in order to better overlap the ferric transient. The characteristic features (*SI Appendix*, Fig. S2 *B* and *C*) of a HS state, i.e., the blue shift and intensity decrease of the K_β_ line around 7,058 eV, and the increase of the K_β_ sideband at 7,043 eV, are clearly reproduced. In Fig. 3, we also show the difference K_β_ steady-state spectra of reference sextet (S = 5/2) and quartet (S = 3/2) minus doublet (S = 1/2) porphyrins (58). Both reproduce the experimental transient well, although they do not allow the identification of the spin of the state populated at 200-fs time delay. Nevertheless, there is no doubt that a state with a higher spin than the ground state is formed.

**Fig. 4.**
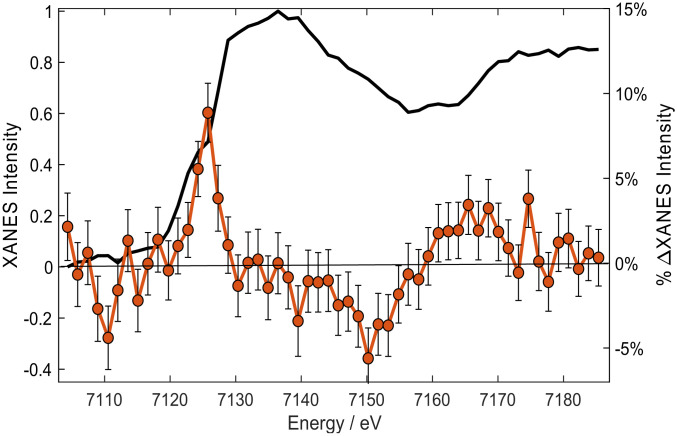
Steady-state XANES ground-state spectrum of ferric Cyt c in room temperature aqueous solutions at pH 7 (black trace) and transient spectrum (excited minus unexcited) recorded 500-fs time delay after femtosecond excitation at 350 nm (brown dots).

The above two observables provided unambiguous evidence of higher spin states being populated upon photoexcitation. As already mentioned, this is what drives doming, which we now identify via its structural XANES signature. Fig. 4 shows the steady-state Fe K-edge XANES of ferric Cyt c (black trace), which agrees with the literature (*SI Appendix*, Fig. S7) (59), along with the transient spectrum (laser-on minus laser-off spectra) 500-fs time delay after 350-nm excitation. The transient shows an increased absorption at the edge energy (∼7,125 eV) followed by a negative signal between 7,130 and 7,160 eV and a positive one beyond. The intensity increase at the edge can either be caused by a reduction of the Fe atom (60), an increase of Fe–N bond distances (as reported for [Fe(bpy)_3_]^2+^; ref. 61), or a combination of both. We rule out photoreduction of the Fe atom based on the fact that its yield is low (<2% for 403-nm excitation) (25, 28), and its rise time is slow (∼5 ps) (25). This is further supported comparing our transient with the difference of the steady-state ferrous minus ferric Cyt c spectra, which strongly deviate from each other (*SI Appendix*, Fig. S8). This type of comparison is commonly used to identify oxidation state changes (42). On the other hand, the transient in Fig. 4 resembles those reported in quasi–steady-state XANES studies of MbCO under continuous wave irradiation (62, 63) and picosecond XANES studies of photoexcited ferrous MbCO (47), MbNO (49), and, more recently, in femtosecond studies of ferrous Cyt c (50). In all these cases, the oxidation state of the Fe atom did not change, and the transient features were caused by the fact that after detachment of the distal ligand from the planar LS hexacoordinated heme, the pentacoordinated heme undergoes doming, i.e., the Fe–N_p_ bonds elongate. In order to highlight the resemblance of the ferric and ferrous heme transients, *SI Appendix*, Figs. S9–S11 compare the ferric Cyt c transient with those for ferrous Cyt c (50), MbNO (49), and MbCO (47). To this purpose, the present ferric transient had to be shifted by approximately −2 eV to best overlap the ferrous Cyt c transient (50). This amount corresponds to the Fe K-edge shift of the ferric and ferrous Cyt c species (59). Thus, apart from this shift, which further confirms that no reduction has taken place, the ferric Cyt c transient strongly resembles those of ferrous species, in particular, Cyt c. Some differences also show up, e.g., the shoulder at ∼7,125 eV in ferrous Cyt c or the larger width of the main feature in MbNO, but the level of agreement is satisfactory and on the same level as that between the various ferrous species. The origin of the deviations may be explained by the quantum yield for ligand photodissociation, which is 100, 80, and 50% for MbCO (64), ferrous Cyt c (23), and MbNO (65), respectively. Thus, two species are generated in the case of ferrous Cyt c and MbNO: one ligated, the other not. This surely contributes to the broadening of the main edge feature in the transient. As a matter of fact, the width of this feature is smallest in ferric Cyt c and MbCO, for which only one species is generated upon excitation. Also of note are that the differences between transients originates from the differences in the ground state XANES spectra of the various species (66). Thus, whether a ferric or ferrous system and regardless of ligand detachment or lack thereof (assumed so far for ferric Cyt c), the main effect on the profile of the XANES transient is that a domed photoproduct is formed. One could conclude from *SI Appendix*, Figs. S9–S11 that the domed species in ferric Cyt c may also be pentacoordinated. While this has been excluded based on transient optical and resonance Raman studies (24, 27), the present results cannot validate it. We thus rely on the conclusions of the optical studies that the ligand does not dissociate (26, 29). However, we conclude that doming via formation of HS states does occur in a ferric heme. This is further supported by a study of the temperature dependence of the Fe K-edge XANES of the hydroxide complex of ferric Mb (Mb^III^OH^−^) between 80 and 300 K (67) for which prior magnetic susceptibilities (68) had established that it is purely LS at 80 K and predominantly HS at 300 K. In their study, Oyanagi et al. (67) found that the peak at ∼7.125 keV in their difference spectra (high temperature minus 80 K spectrum) clearly increases with temperature and reproduces the same trends as the magnetic-susceptibility measurements. From the above considerations, it is fair to conclude that the effect determining the overall shape of the transients in Fig. 4 and *SI Appendix*, Figs. S9–S11 is doming, via population of an IS or HS state.

**Fig. 5.**
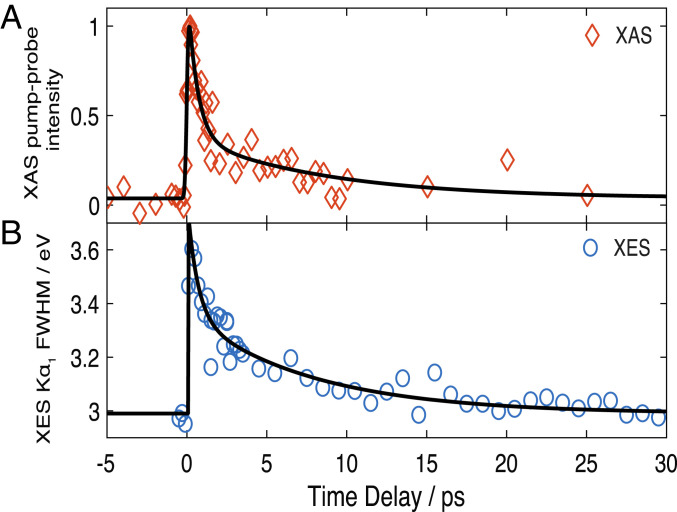
Temporal evolution of the transient XANES and XES signals. (*A*) Intensity of the XANES signal at 7,125.3 eV. (*B*) FWHM extracted from an asymmetric Voigt fit of the K_α1_ line at 6,405 keV (*SI Appendix*, section 6 and Fig. S6). The black trace represents the fit using a function with an IRF-limited rise (∼140 fs for the XAS and ∼150 fs for the XES) and a biexponential decay. The fit parameters (time constants and preexponential factors) are given in *SI Appendix*, Table S1.

In summary, both the K_α_ and K_β_ XES signals via an electronic signature and the XANES signal, via a structural signature, point to formation of IS or HS states within the first picosecond. Further insight into the relaxation dynamics is provided by the temporal traces of the signals, which are shown in Fig. 5*A* for the XANES signal at 7.125 keV (maximum amplitude) and Fig. 5*B* for the FWHM of the fit of laser-on K_α1_ lines. Both traces exhibit an instrument response function limited rise (≤150 fs; *SI Appendix*, section 2), followed by a decay that is best fit using a biexponential function convoluted to the Gaussian instrument response function (*SI Appendix*, section 5). They both yield consistent time constants of ∼600 fs and ∼8 ps, in very good agreement with previous optical TA studies (Table 1) (26). Considering that the XES is a pure electronic signature and is not sensitive to vibrational effects, we can conclude that these times reflect an electronic relaxation among spin states. However, the preexponential factors differ between the XANES and the XES signals (Table 1). This is due to the fact that the XANES transient signal at 7.125 keV reflects a structural signature, whose intensity is not simply linear with the Fe–N bond elongation (see e.g., ref. 61). Although the latter is largest for the S = 5/2 state, the S = 3/2 state surely contributes to the signal at early times but is not distinguishable in the XANES transient. From the kinetics, we can conclude that the ∼600-fs time scale is due to decay of the S = 3/2 state, while the ∼8 ps is due to the S = 5/2 state relaxing back to the ground state (S = 1/2). Further supporting this conclusion is that in Fig. 2*B* and *SI Appendix*, Fig. S4, *Bottom*, the positive peak at 6,406 eV and the negative one at 6,404 eV of the K_α1_ transient show somewhat different evolutions. By integrating the signal of the positive and negative peaks and plotting them as a function of time (*SI Appendix*, Fig. S12*A*), we recover two different kinetic traces (*SI Appendix*, Fig. S12*B*). Despite the few data points, it can be seen that the negative part shows a prompt rise with a biexponential decay, comparable to Fig. 5, while the positive signal shows a slower rise and a monoexponential decay. Fitting these kinetic traces with multiexponential functions yields time constants comparable to those derived from Fig. 5. In particular, the short-decaying component (∼700 fs) of the negative signal is quite close to the rise of the positive signal, suggesting a cascade process in which an intermediate state feeds population into the HS state. This analysis also reveals hitherto unknown spin information contained in the K_α_ spectra, which deserves further investigation for a more general class of systems.

**Table 1.** Comparison of the rise (τ_r_) and decay times (τ_i_) of the transient XANES reported in this work with previous ultrafast optical studies carried out by fluorescence up-conversion spectroscopy (69) and UV-visible TA spectroscopy (26)

Method (pump wavelength)	τ_r_	τ_1_ (a_1_)	τ_2_ (a_2_)	τ_3_ (a_3_)
XANES (350 nm) (this work)	0.14 ± 0.03	0.61 ± 0.2 (0.70)	—	8.7 ± 4 (0.30)
K_α_ XES (400 nm) (this work)	0.15 ± 0.03	0.63 ± 0.12 (0.50)	—	7.8 ± 4 (0.50)
Ultrafast fluorescence of the LUMO (400 nm) (69)	≤0.04	≤0.04		
Optical TA (530 nm) (26)	0.22 ± 0.02	0.70 ± 0.09	3.4 ± 0.3	11 ± 2

The optical fluorescence monitors the rise and the decay of the LUMO of the porphyrin macrocycle (i.e., the Q-bands), while the TA monitors the return of the population to the HOMO ground state. Both methods are sensitive to π−π* transitions of the porphyrin. The X-ray signal is sensitive to the electron density on the Fe atom, and therefore it monitors its redistribution after photoexcitation. The a_i_s are the preexponential factors. The dashes respresent time scales that were not observed in the corresponding experiments. The error bars are based on CIs of the fit. All time constants are in picoseconds.

**Fig. 6.**
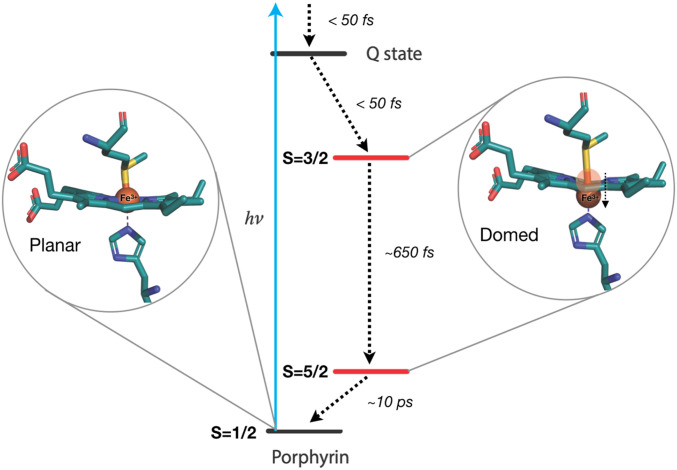
Schematic representation of the relaxation cascade following UV-visible excitation of ferric Cyt c. The energy levels are arbitrarily placed. Excitation of the system leads to an ultrafast population and decay of the Q-state (the porphyrin LUMO), as established by fluorescence up-conversion studies (70); the IS S = 3/2 state is promptly populated and decays in ∼650 fs to the HS S = 5/2 state, which then decays back to the ground state in ∼10 ps.

The comparison of the time constants derived here with those from optical experiments (Table 1) provides a complete picture of the photocycle of ferric Cyt c. The optical fluorescence monitors the rise and decay of the porphyrin LUMO (69), which both occur at ultrafast time scales (<50 fs), and the decay was attributed to relaxation to metal spin states. The optical TA experiments also probe the porphyrin π−π* transitions, but they monitor the recovery of the ground state (HOMO) for which time constants of ∼700 fs, ∼3.5 ps, and ∼11 ps had been retrieved (26). On the other hand, the XANES and XES observables selectively sense the electronic distribution on the Fe atom. Thus, the prompt rise of the X-ray time traces reflects rapid (<50-fs) formation of an excited metal spin state, most likely the S = 3/2 state, via decay of the LUMO (69) and with no change of the metal oxidation state. The subsequent decay of the X-ray signal occurs on two timescales (∼600 fs and ∼8 ps), which are identical to the first and third components reported in the optical TA (Table 1). Our results show that these times are not due to a hot heme vibrational relaxation, as previously claimed (7, 24, 26, 38) (XES is not sensitive to thermal effects), but rather to a population relaxation through spin states. The X-ray kinetic traces can thus be explained by a cascade via a promptly formed S = 3/2 state that relaxes in 600 to 700 fs to the S = 5/2 state, which then returns to the initial S = 1/2 ground state in ∼8 ps, as schematically shown in Fig. 6. Of course, a parallel vibrational cooling channel in the return to the initial state is also possible, but the close correspondence between the time constants of the present study and the optical ones (Table 1) supports the conclusion that the ∼600-fs and ∼8-ps time scales are due to an electronic relaxation cascade. In this respect, the intermediate (∼3.5-ps) component reported in most optical studies (7, 24, 26) is likely due to a thermal relaxation within the HS state. We also performed a triexponential function to fit the X-ray time traces (Fig. 5) and did retrieve an additional ∼3-ps intermediate component, but its weight was weak and uncertainty large, while the first and third components changed neither in decay times nor in relative weights. We therefore hold the biexponential fit of the X-ray data as physically more meaningful. Further support that the relaxation is predominantly electronic, i.e., with minimal parallel thermal relaxation channels, comes from the estimates of the photolysis yield from the XANES transient signal compared with the photoexcitation yield based on the experimental parameters (energy/pulse, focal spot, concentration, absorption coefficient, sample thickness, etc.) presented in *SI Appendix*, section 4. These estimates agree to within a factor of 2, which bearing in mind that scattering and reflection losses of the laser pump beam are not considered, confirm that nearly all photoexcited species relax by an electronic cascade via spin states.

The above considerations can be generalized to other ferric hemoproteins. Indeed, they support previous optical studies that spin states are involved in the relaxation of photoexcited ferric MbCN (34), MbN_3_ (35), and metMb (39). This is further reflected by the rather similar kinetic behavior of all these systems upon excitation of either the Q- or Soret-bands (*SI Appendix*, Table S1). Three time scales emerge, subpicosecond, picosecond, and few picoseconds, which vary somewhat from system to system due to the different distal ligands but show a good overall agreement. Thus, the evidence is compelling that heme relaxation proceeds via a cascade through spin states, leading to heme doming without ligand dissociation in all of the thus-far investigated ferric hemes. However, the intermediate τ_2_ ∼3-ps time scale in *SI Appendix*, Table S1 reflects vibrational relaxation in the HS state and is similar to the value reported for various heme proteins both ferrous and ferric (24, 37, 71), as well as [Fe(bpy)_3_]^2+^ (72, 73).

Based on the present and previous results, doming is a deformation that is present in all heme proteins, whose role is well established in the respiratory function of ferrous Mb and Hb. The question arises about its biological significance in ferric hemes, as functionally diverse as Cyt c, metMb, MbCN, MbN_3_, MbOH, and oxy-FixLH. Doming, i.e., HS states, may play a role if their energies with respect to the LS states are at kT, where k is the Boltzmann factor and T the temperature. Quite remarkably in ferric MbN_3_, it was found that the photocycle is identical whether one excites the Q-band in the green or the N_3_ stretch mode in the region of 2,000 cm^−1^, and the occurrence of a HS state was invoked (35). This suggests that the HS state is low-lying, in line with the above-discussed results on MbOH (67).

## Conclusions

In summary, in the present study, we have combined element-specific, structure-sensitive (XANES), and spin-sensitive (XES) methods to study the relaxation of photoexcited ferric Cyt c. We have shown that its photocycle is entirely due to a cascade among spin states that occurs via a prompt decay of the LUMO into the S = 3/2 IS state, followed by a further decay in ∼600 to 700 fs into the HS S = 5/2 state, which causes doming. The S = 5/2 state decays back to the ground S = 1/2 state in ∼10 ps. Except for the time scales that differ, this process is identical to the SCO dynamics in Fe–polypyridine complexes (74, 75). The occurrence of doming in heme proteins is therefore not limited to the ferrous forms, but it also includes ferric ones. The fact that doming is present in very diverse ferric hemes raises the question of its biological role and calls for further studies.

## Methods

A brief description of experimental methods is given here. Additional details of the sample preparation, experimental setup, and analysis are provided in *SI Appendix*. The experiments were carried out at SwissFEL (femtosecond XANES and femtosecond XES) and European XFEL (femtosecond XES). A 4 mM Cyt c solution in a phosphate buffer, delivered by a liquid jet, was photoexcited into the Soret-band of the porphyrin by s-polarized (perpendicular to the X-rays) pump pulses in the region of 350 to 400 nm, at a fluence of <22 mJ/cm^2^ (*SI Appendix*, Figs. S13 and S14). The XANES was recorded by scanning the monochromatized X-ray beam across the Fe K-edge (7.12 keV), while the XES was obtained by spectrally dispersing the emission of the sample, irradiated by an ∼8-keV pink beam, with a von Hamos spectrometer to obtain both the Fe K_α_ and K_β_ emission lines. Both sets of measurements were repeated for different pump-probe delays in order to capture the full relaxation pathway of the system and its corresponding kinetic traces.

## Supplementary Material

Supplementary File

## Data Availability

Raw data were generated at SwissFEL and the European XFEL large-scale facilities, and due to the nature and quantity of data produced, are available upon request. All derived data supporting the findings of this study and the corresponding analysis scripts are available through the Open Science Repository (https://osf.io/qn4vh/?view_only=956be317ca504caf8c7158b69a9a3dec).
