# Supplementary material for: Spin cascade and doming in ferric hemes: Femtosecond X-ray absorption and X-ray emission studies
Source: Proc Natl Acad Sci U S A. 2020 Aug 26;117(36):21914–20. doi: 10.1073/pnas.2009490117 (PMC7486745; doi:10.1073/pnas.2009490117)
Supplement: Supplementary File [file pnas.2009490117.sapp.pdf]

Supplementary Information

Spin cascade and doming in Ferric Haems: Femtosecond X-ray Absorption and X-ray Emission Studies

Camila Bacellar[1], Dominik Kinschel[1], Giulia F. Mancini[1], Rebecca A. Ingle[1], Jérémy Rouxel[1], Oliviero Cannelli[1], Claudio Cirelli[2], Gregor Knopp[2], Jakub Szlachetko[3], Frederico A. Lima[4], Samuel Menzi[2], Georgios Pamfilidis[2], Katharina Kubicek[4], Dmitry Khakhulin[4], Wojciech Gawelda[4,5], Angel Rodriguez-Fernandez[4], Mykola Biednov[4], Christian Bressler[4], Christopher A. Arrell[2], Philip J. M. Johnson[2], Christopher Milne[2] and Majed Chergui[1]

[1] Ecole Polytechnique Fédérale de Lausanne (EPFL), Laboratoire de Spectroscopie Ultrarapide, ISIC and Lausanne Centre for Ultrafast Science (LACUS), CH-1015 Lausanne, Switzerland

[2] SwissFEL, Paul-Scherrer-Institut (PSI), 5232 Villigen PSI, Switzerland

[3] Institute of Nuclear Physics, Polish Academy of Sciences, 31-342 Kraków, Poland

[4] European XFEL, Holzkoppel 4, D-22869, Schenefeld, Germany

[5] Faculty of Physics, Adam Mickiewicz University, ul. Uniwersytetu Poznanskiego 2, 61-614 Poznan, Poland

Table of contents

1. Sample preparation
2. Experimental Methods
    a. SwissFEL Measurements
    b. European XFEL Measurements
3. Data Collection and Analysis
4. Estimating the photolysis yield
5. Time Scans
6. Fit of line shapes
7. Tables
8. Figures

1. Sample preparation

The ferric redox state of the sample was prepared from horse heart Cytochrome c (Cyt c), purchased from Lee Biosolutions, dissolving it in a pH7 phosphate buffer solution (30 mM), to obtain a final concentration of 4 mM. The oxidation state of the sample was verified by

www.pnas.org/cgi/doi/10.1073/pnas.2009490117

measuring the steady-state UV-Visible (1) and X-ray (2) absorption spectra, which clearly differ from those of ferrous Cyt c. The latter was prepared by adding a molar excess of sodium dithionate ($Na_2S_2O_4$) to reduce the sample.

2. Experimental Methods

The X-ray absorption (XAS) and X-ray emission spectroscopy (XES) experiments were carried out at the Alvra end-station of the swiss Free Electron Laser (SwissFEL) at the Paul-Scherrer-Institut (Villigen).(3) The XES experiments were then repeated at the FXE end-station of the European-XFEL (Eu-XFEL, Hamburg).(4)

a) <u>SwissFEL measurements</u>:

The sample was delivered to the interaction region by a cylindrical liquid jet of 100 µm diameter, running in a closed loop. The jet was pumped into the chamber by an HPLC pump and out by a peristaltic one. Right at the chamber exit, a mobile UV-visible spectrometer provides an on-line monitoring of the sample integrity. No sample damage was observed during the measurements.

The pump pulse was generated by an 800 nm Ti:Sapphire amplified laser system coupled to an optical parametric amplifier, running at 12.5 Hz (half the repetition rate of the X-ray pulses) and yielding ~60 fs/350 nm laser pulses to excite the sample. This pump wavelength was chosen in order to match the penetration depth of the laser to the jet thickness while providing high energy excitation of the haem group. The fluence dependence of the XANES transient signal at 7125 eV and 500 fs time delay after excitation at 350 nm is shown in figure S13 and it exhibits a linear response up to about 20 mJ/cm^2. The measurements were carried out at a fluence range of 14 - 22 mJ/cm^2 at the sample position. Such fluences are comparable to those used in the ps and fs Fe K-edge absorption studies of ferrous myoglobins and ferrous Cyt c.(5-7) The jet speed was such that the sample was completely replenished between consecutive pairs of laser pump/X-ray probe pulses.

The laser and X-ray beams hit the sample in a nearly collinear geometry. The X-ray beam was focused into a 30x40 μm^2 (FWHM) spot by a pair of Kirkpatrick-Baez mirrors. The laser focus was in the order of 100x130 μm^2 (beam waist). Tune-able monochromatic x-rays in the 7.1-7.2 keV range were used for the Fe K-edge absorption measurements, and were generated by scanning the FEL electron beam and the Si(111) monochromator central energies. For the XES

experiment, the SASE beam was used and the energy set to 8 keV, away from any Fe shape resonances.

The relative time delay between the laser and x-ray pulses was tuned by a delay stage and scanned in the region of -5 to +20 ps. The instrument response function (IRF) of the experiment was obtained from the rise time of the Fe XANES signal and confirmed by a separate measurement of the rise time of a YAG crystal using an X-ray pump/optical probe combination,(8) in which the X-ray pulses are used to pump a YAG crystal, changing its reflectivity due to the creation of free charge carriers. The transient optical transmission of the 350 nm probe though the YAG is then detected by a diode and used to measure the X-ray/optical cross correlation. The IRF is ~140 ± 30 fs (FWHM), with the uncertainty being mainly due to the intrinsic jitter in arrival time between optical and X-ray pulses.

The XANES spectra were recorded by a Be shielded Si diode in total fluorescence yield (TFY) mode at approximately 90º from the incident X-ray beam and normalized by the incoming X-ray pulse intensity (I_0), measured by the scattering from a thin target placed about three meters upstream in the beam path. The FEL was running at a repetition rate of 25 Hz, twice that of the pump laser, such that the transient data were obtained by subtracting consecutive laser-on from laser-off shots. The XES data were measured by a spectrometer based on the von Hamos geometry.(9) Two cylindrical Ge(111) crystal analyzers were used to collect the Fe $K_{\alpha 1}$/$K_{\alpha 2}$ fluorescence and focus it onto a 4.5 Megapixel 2D Jungfrau detector. The experiments were carried on a 600 mbar He atmosphere to maximize X-ray transmission and minimize X-ray scattering noise.

b) European XFEL measurements:

A very similar set-up to the above was utilized for recording the XES data at FXE. The sample delivery system is essentially identical and the jet thickness was also kept constant (100 µm) between measurements in the different facilities. While an on-line monitoring of the sample was not implemented at European XFEL, aliquots of the ferric Cyt c samples used were collected every couple of hours and their UV-Visible absorption spectra did not show signs of either laser of x-ray damage.

X-ray probe pulses with an energy of 9.3 keV were used. The X-ray delivery pattern consisted of pulse trains arriving at 10 Hz repetition rate, each train containing 128 pulses at a repetition rate of 564 kHz (i.e. 1280 pulses/s). The frequency-doubled output pulses (400 nm) of an

amplified custom NOPA based laser system (10) were used to pump the sample. The pump laser was focused down to 90 x160 μm^2 (FWHM) while the x-rays were focused to 10x13 μm^2 (FWHM). The pump fluence was in the order of 9-10 mJ/cm^2, chosen to maximize the S/N while still within the linear regime, as shown in Figure S14. The IRF was determined by the rise time of a $[Fe(bpy)_3]^{2+}$ XES pump-probe signal to be ~150 fs (FWHM), mainly due to the intrinsic jitter between the arrival time of the laser and x-ray pulses and the group velocity mismatch between laser and x-ray pulses over the 100 μm sample thickness.

The data were acquired with laser-off and laser-on spectra recorded alternatively at 5 Hz. The XES signal was collected by a 16-crystal von Hamos spectrometer and detected by a Jungfrau 1M detector at 10 Hz, integrating all the signal within a pulse train by the detector. Both K_α and K_β spectra were acquired simultaneously, such that eight 500 mm radius Si(531) cylindrically bent analyzers were used for the K_β detection and another eight Ge(111) crystals were used for $K_{\alpha1}/K_{\alpha2}$ lines. While the sample/spectrometer setup were not enclosed in a vacuum chamber, a He bag was placed between the sample and spectrometer to minimize the XES attenuation by air. The following data analysis and treatment were identical to those performed on the SwissFEL results.

3. *Data Collection and Analysis*

XANES spectra were acquired at SwissFEL over 4000 FEL shots per energy point (2000 laser-on, 2000 laser-off) and normalized by the incoming X-ray pulse intensities on a single shot basis. A threshold was set such that the low intensity X-ray shots, due to the intrinsic large flux fluctuations of monochromatized SASE beams, are discarded in order to improve the S/N of the measurements. Since the laser focus was substantially larger than the X-ray spot size, and presented a high shot-to-shot fluence stability, we assume uniform excitation across the entire probed volume. Three complete XANES spectra were averaged together, resulting in the data presented in this work. The energy axis was calibrated against a reference Cyt c spectrum from the literature (Figure S2).(2)

4. *Estimating the photolysis yield:*

The excitation fraction was estimated based on the reference Cyt c Fe^{2+} XANES spectra of ground state (GS) and excited state (ES) provided in ref. (7). By assuming the XANES spectrum at any given time to be a linear combination ES and GS contribution ($XANES(t) = \alpha(t) * ES +$

$(1-\alpha(t)) * GS$)), we can estimate the fraction of the excited state species in the pump-probe signal, by minimizing the following expression:

$$\Delta XANES(t) - \alpha(t) * (ES - GS) \qquad (1)$$

At 500 fs, the photoexcitation fraction $\alpha \approx 50\%$. Since at this time delay 25% of the ES population has already decayed, we can extrapolate the excited state fraction immediately after photoexcitation to be in the order of $\alpha(t=0) \approx 67\%$.

This number can be compared with the expected photoexcitation yield based on the excitation conditions. Considering the laser fluence (22 mJ/cm^2), the absorption cross section (1.9×10^4 $M^{-1}cm^{-1}$) and the number of molecules in the irradiated volume (~10^{13} molecules), approximately ~1.35 photons are absorbed per molecule, which would put the photoexcitation yield to 100%. However, this estimate does not consider losses due to scattering and reflection from the liquid jet, which was round shaped, i.e. such losses are non-negligible but could not be quantified. We therefore believe that the two estimates are in good agreement, i.e. that the X-ray signal reflects the response of the entire population of excited species. The above numbers compare well with the ~3 photons/molecule used in the fs XAS and XAS study of ferrous cytochrome c(7) and the ~2.2 photons/molecule, used in the XAS study of MbCO.(6)

5. *Time Scans*

The temporal trace is an average of three individual scans collected over several thousands FEL shots at the maximum of the pump-probe signal at 7125.3 eV (Figure 2). As mentioned above, the instrument response function (IRF~140 fs) was determined from pump-probe scans on a YAG substrate and it agrees with the rise time obtained by the fit of the XANES traces.

To fit the time traces, a bi-exponential decay function was used:

$$D_n(t) = \frac{1}{2} * e^{-\frac{1}{t_n}\left(t-t_0-\sigma^2/t_n\right)} * \left[1 + erf\left(\frac{t-t_0-\sigma^2/t_n}{\sqrt{2}\sigma}\right)\right] \qquad (2)$$

$$I(t) = a_1 * D_1(t) + a_2 * D_2(t) + C \qquad (3)$$

where $I(t)$ is the intensity of the pump-probe signal at a given time-delay, t; t_0 is time-zero; σ is the FWHM of the IRF, a_1, a_2 are the pre-exponential factors to the exponential decay time constant with t_1, t_2 time constants, respectively and C is a constant offset.

The above equation delivers the best fit with the parameters reported in Table S1. A three-exponential fit was also attempted but the first and third components were highly correlated and

a very small amplitude was observed for the intermediate one, confirming that the bi-exponential model is the best suited to describe the results.

6. Fit of line shapes

The fits of the $K_{\alpha 1}$ XES data were performed using an asymmetric pseudo-Voigt function, as described in detail in ref. (11). Briefly, the XES line shape ($V(x)$) can be fit by equation (4) plus an additional linear offset (eq. 6) to correct for non-uniform background.

$$V(x) = (1-m)\sqrt{\frac{4\ln(2)}{\pi\omega_{(x)}^2}}exp\left[-\left(\frac{4\ln(2)}{\omega_{(x)}^2}\right)x^2\right] + m\left(\frac{1}{2\pi}\right)\left(\frac{\omega_{(x)}}{\left(\omega_{(x)}/_2\right)^2 + 4x^2}\right) \quad (4)$$

Where

$$\omega_{(x)} = \frac{2\omega_0}{1+exp(-a(x-x_c))} \quad (5)$$

and

$$offset\ = slope * x + C \quad (6)$$

The physically relevant parameters are the FWHM (ω_0) peak position in energy (x_c). The asymmetry is introduced with the energy dependence of the FWHM, $\omega_{(x)}$, and expressed by the asymmetry factor a. It's worth noting that for large asymmetry parameters the fit parameter ω_0 can differ significantly from the actual FWHM of the line shape,(11) however for the fits presented in this work the numerically calculated FWHM agrees with the extracted fit parameter ω_0 within less than 2% deviation, due to the small asymmetric parameter of the XES line shape.

The fits were repeated for the $K_{\alpha 1}$ XES obtained at different time delays and the evolution of the FWHM (ω_0) as a function of time is displayed on Fig. 3. The other fit parameters did not show any appreciable trend beyond the precision of the fit procedure.

Table S1: Comparison of the rise (τ_r) and decay times (τ_i) reported in the present system and different ferric haem proteins, without precluding of their attribution. All time constants are in picoseconds.

**Species**	τ_r	τ_1	τ_2	τ_3
Ferric Cyt c (this work)	0.15	0.62±0.06		8.2±4
Ferric Cyt c (1)	<0.05	0.7±0.09	3.4±0.3	11±2
MetMb (12)	<0.08	0.51	1.14	4.8
MbCN (13)	< 0.05	0.3		3
MbN_3 (14)			2.4	18

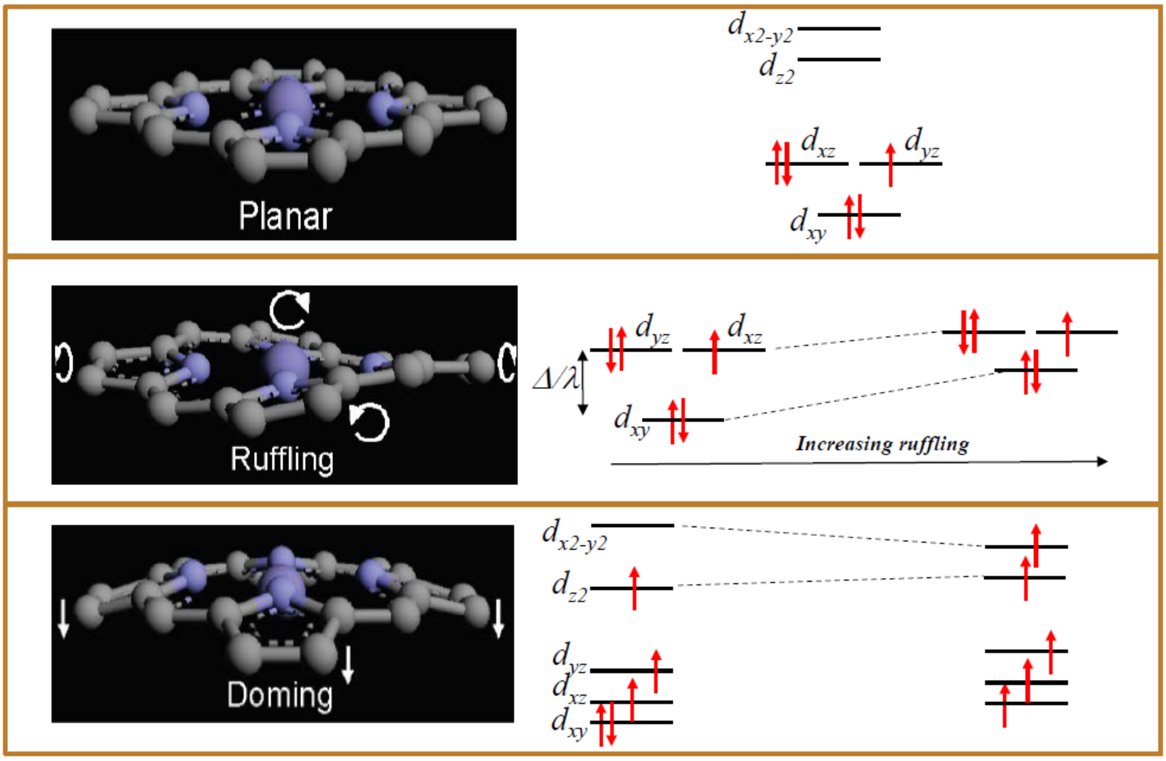

Figure S1: Different haem conformations (reproduced from ref. (15)) and their associated orbital structure. Δ/λ is the axial ligand field parameter, with Δ being the ligand-field splitting and λ the spin-orbit coupling parameter.

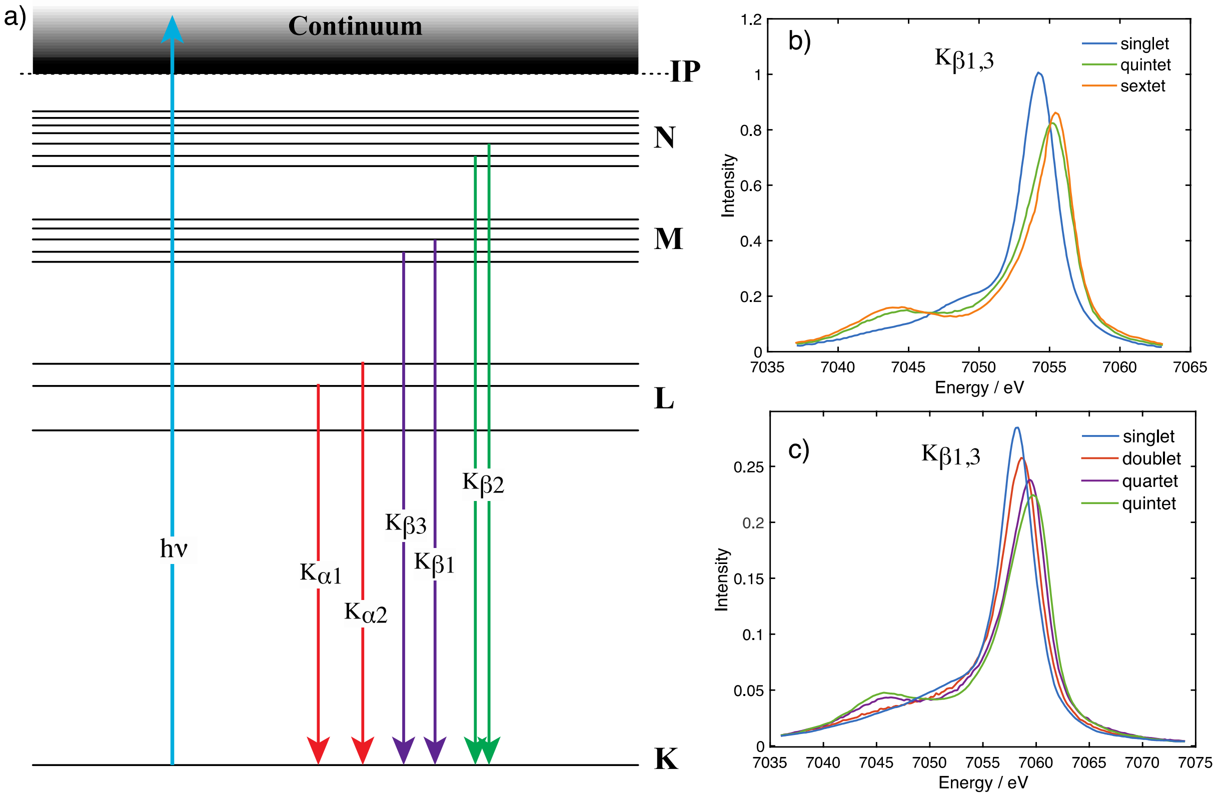

Figure S2: (a) Various emission lines that appear upon creation of a hole in the K-shell of an atom. The various orbitals for L, M, etc. correspond to the sub-shells L_1, L_2, L_3 and M_1, M_2, M_3, M_4, M_5, etc.; b) Kβ emission of model porphyrin compounds of various ground state spins from ref. (16): singlet (Fe^{II}(TPP)$(Py)_2$), quintet (Fe^{II}(TPP)$(THF)_2$) sextet (Fe^{III}(TPP)Cl, TPP = tetraphenylporphyrin, Py = pyridine, THF = tetrahydrofuran). Note the blue shift and intensity decrease of the main line around 7055 eV and the intensity increase of the $K_{\beta'}$ line around 7040-7045 eV; c) same for other compounds, from ref. (17): singlet ($Fe^{II}(Bpy)_3$), doublet ($Fe^{III}(Bpy)_3$), triplet (Fe^{II}(PTC)), quartet (Fe^{III}(PTC)Cl), quintet ($Fe^{II}(Phen)_2(NCS)_2$, Bpy= bipyridine, PTC= phthalocyanine, Phen= phenanthroline).

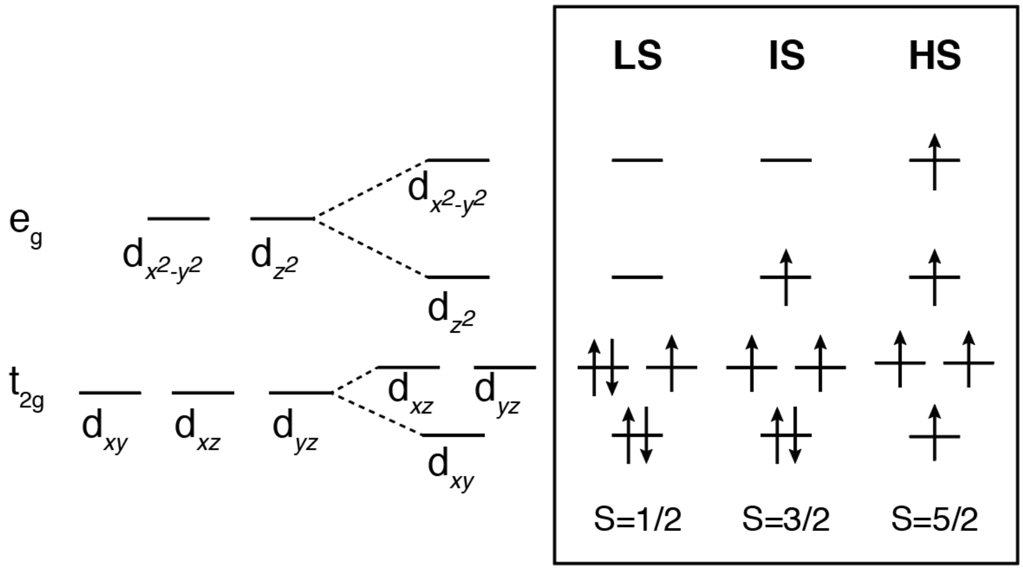

Figure S3: Different spin states: Low spin (LS), intermediate spin (IS) and High spin (HS) state that arise in a ferric system with ligand field splitting.

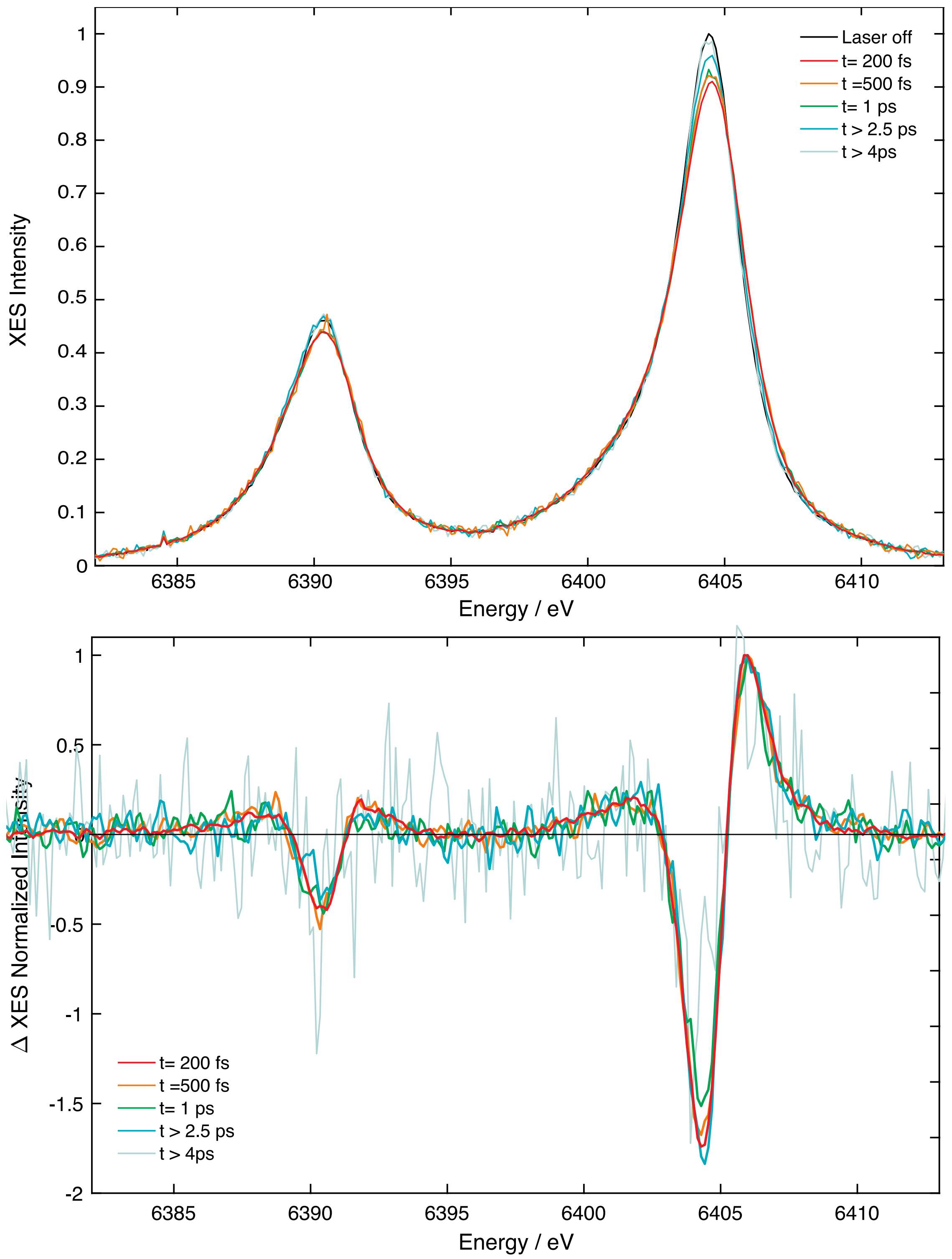

Figure S4: (Top) laser-off and laser-on Kα emission spectra of Cyt c ($K_{\alpha 1}$ around 6044 eV and $K_{\alpha 2}$ around 6390 eV). The laser-on spectra were obtained with 400 nm pump excitation. **(**Bottom) Normalized to the maximum of the transient signal at 6406 eV at 200 fs, 500 fs, 1 ps, > 2.5 ps and > 4 ps (red, orange, green, blue and grey lines) after 400 nm excitation.

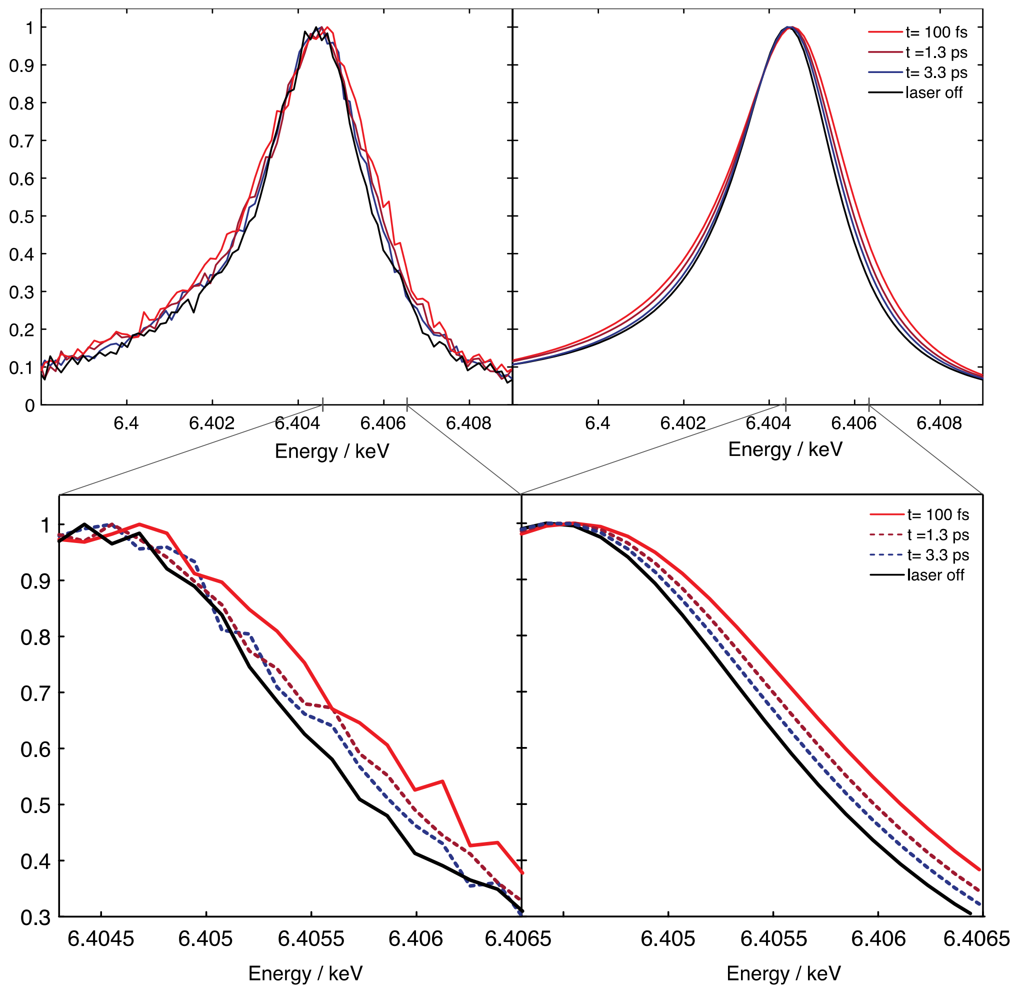

Figure S5: (Top) zoom into the experimental $K_{\alpha 1}$ line (left panel) showing the broadening with time, as well as the fitted line shapes (see § S6 and figure S6). (Bottom) zoom into the blue wing of the line.

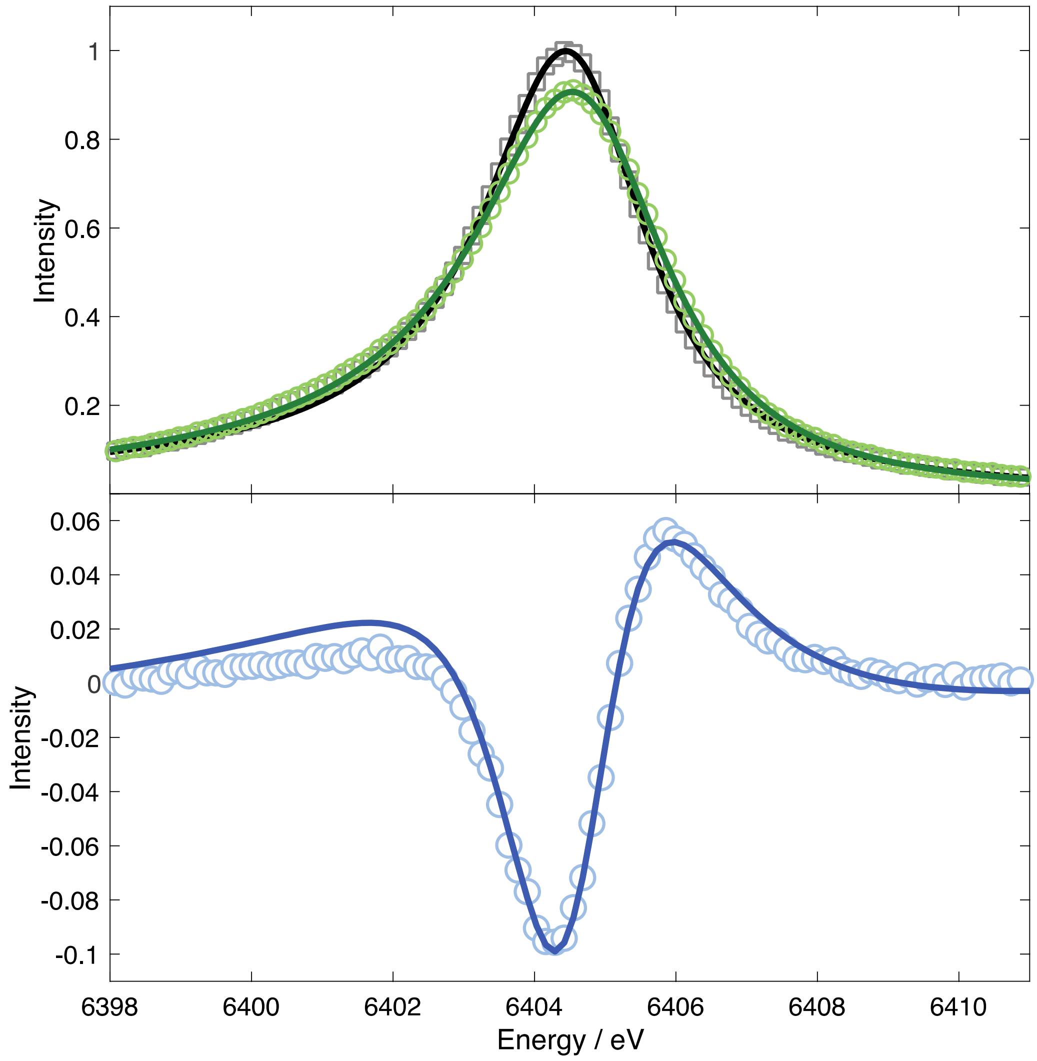

Figure S6: (Top) laser-off Fe $K_{\alpha 1}$ (6405 eV) line (grey squares) and laser-on line (green circles) at 200 fs time delay, along with their fits using an asymmetric pseudo-Voigt function (black and green trace, respectively). (Bottom) The transient XES at 200 fs (light blue dots) and fit of the difference from two asymmetric Voigt line shapes (blue line).

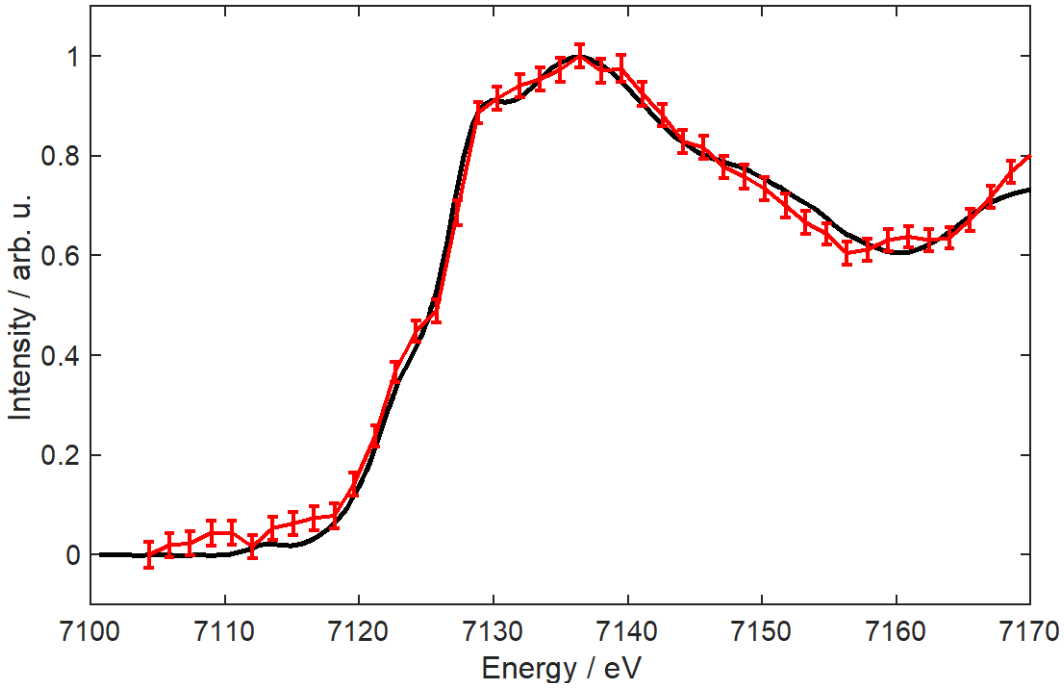

Figure S7: Steady-state XANES spectrum of ferric Cyt c in a room temperature aqueous solution at pH7 recorded at the ALVRA beamline of the swissFEL (red dots), compared to a reference spectrum from the literature (black trace).(2)

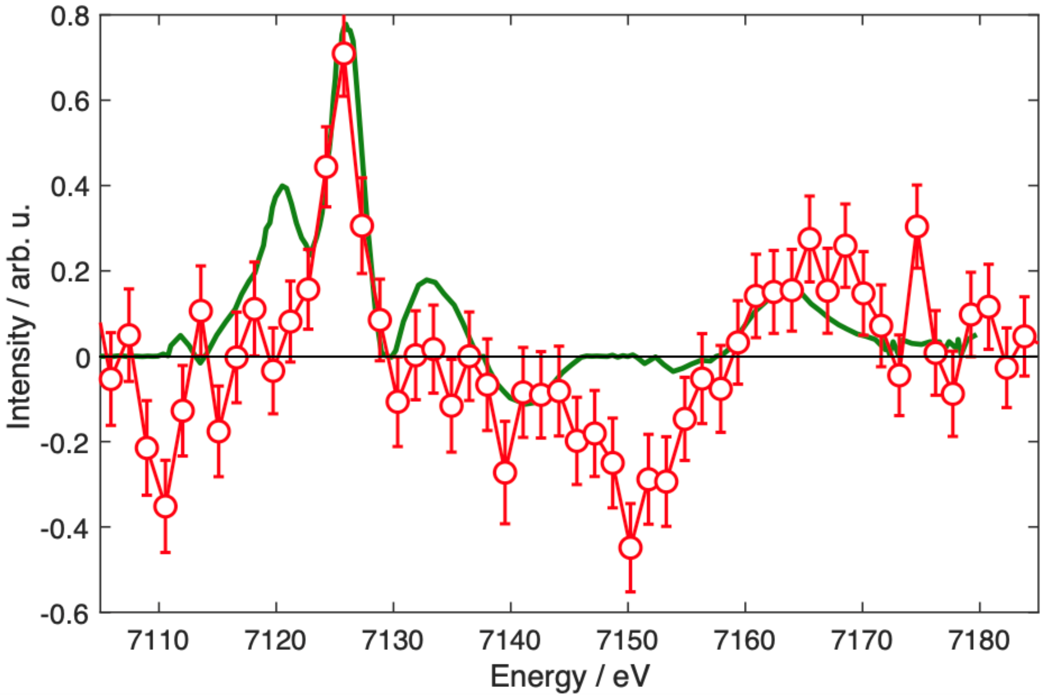

Figure S8: Comparison of the transient Fe K-edge XANES of ferric Cyt c at 500 fs after excitation at 350 nm (red dots), with the difference of the steady-state XANES spectra of ferrous and ferric Cyt c (green).(2) The poor overlap between the traces rules out the occurrence of photoreduction in the present study.

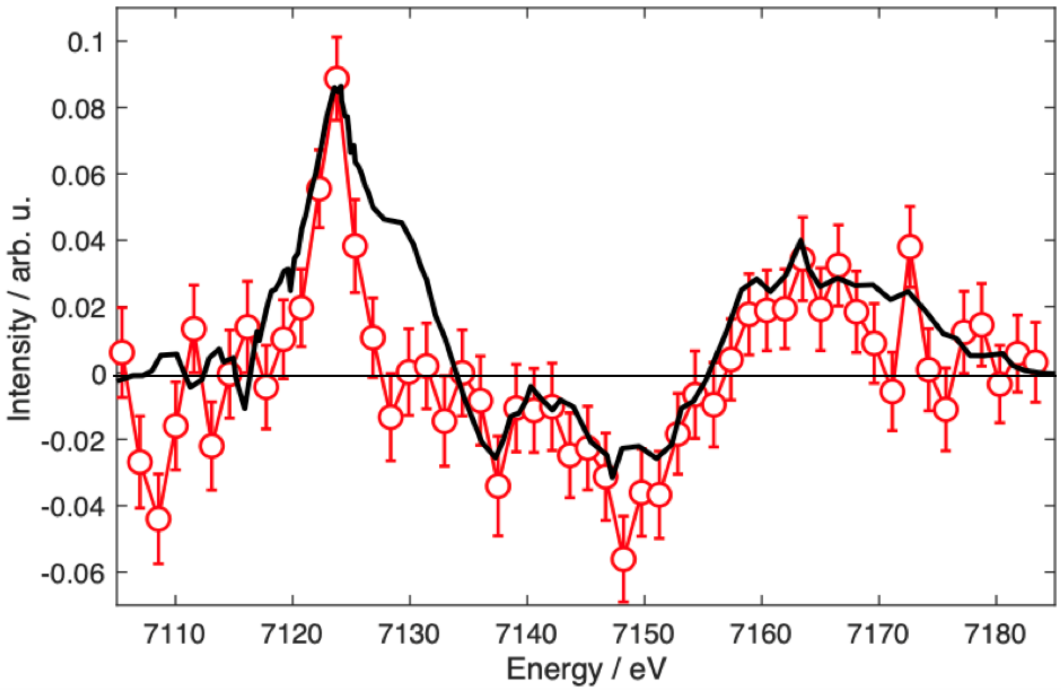

Figure S9: Transient Fe K-edge XANES spectrum of Ferric Cyt c at 500 fs after excitation at 350 nm (red dots), compared to the transient spectrum (black trace) for Ferrous Cyt c obtained by subtracting the laser-on minus laser-off spectra reported in reported in ref. (7). The ferric transient has been red-shifted by ~2 eV to best overlap the ferrous one.

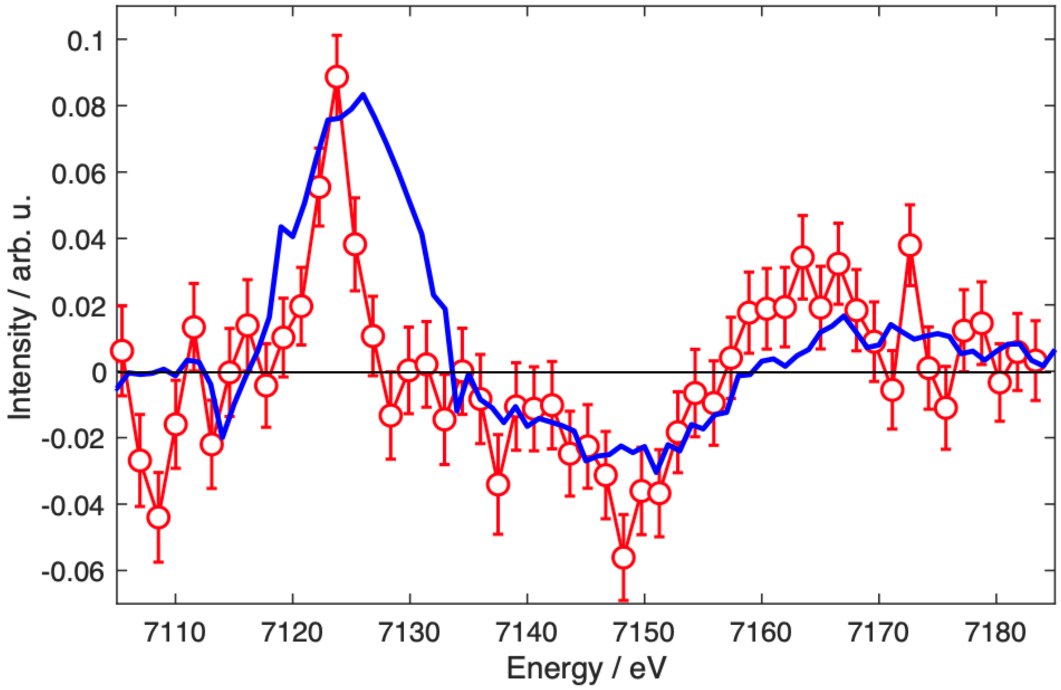

Figure S10: Transient Fe K-edge XANES spectrum of Ferric Cyt c at 500 fs after excitation at 350 nm (red dots), compared to the transient spectrum (blue trace) for MbNO (reproduced from ref. (5)).

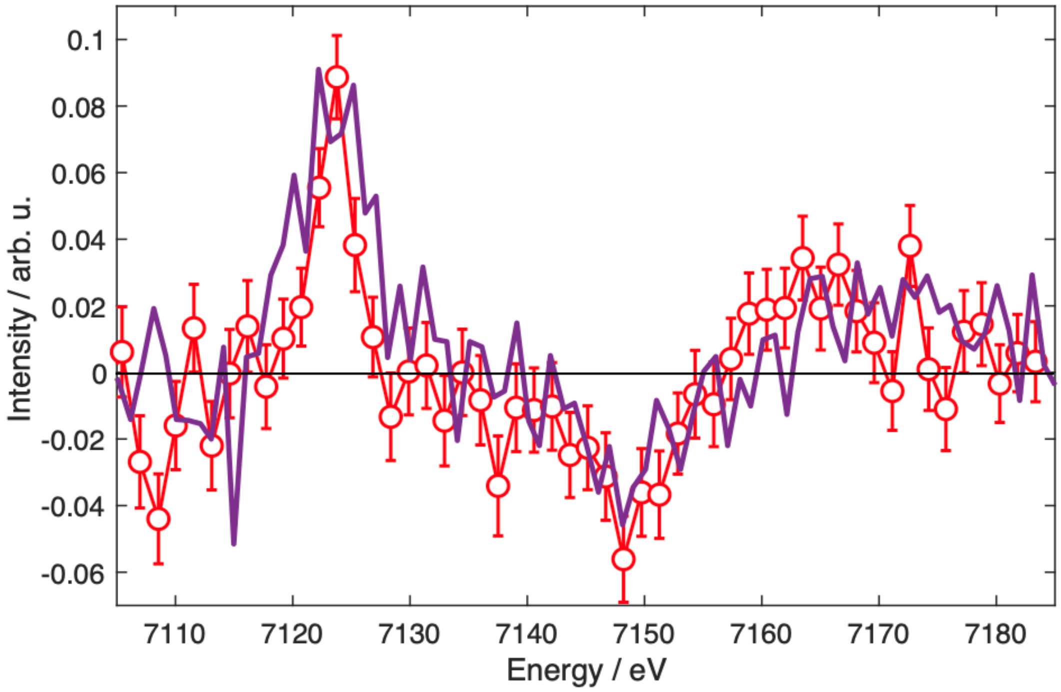

Figure S11: Transient Fe K-edge XANES spectrum of Ferric Cyt c at 500 fs after excitation at 350 nm (red dots), compared to the transient spectrum (purple trace) for MbCO (reproduced from ref. (18)).

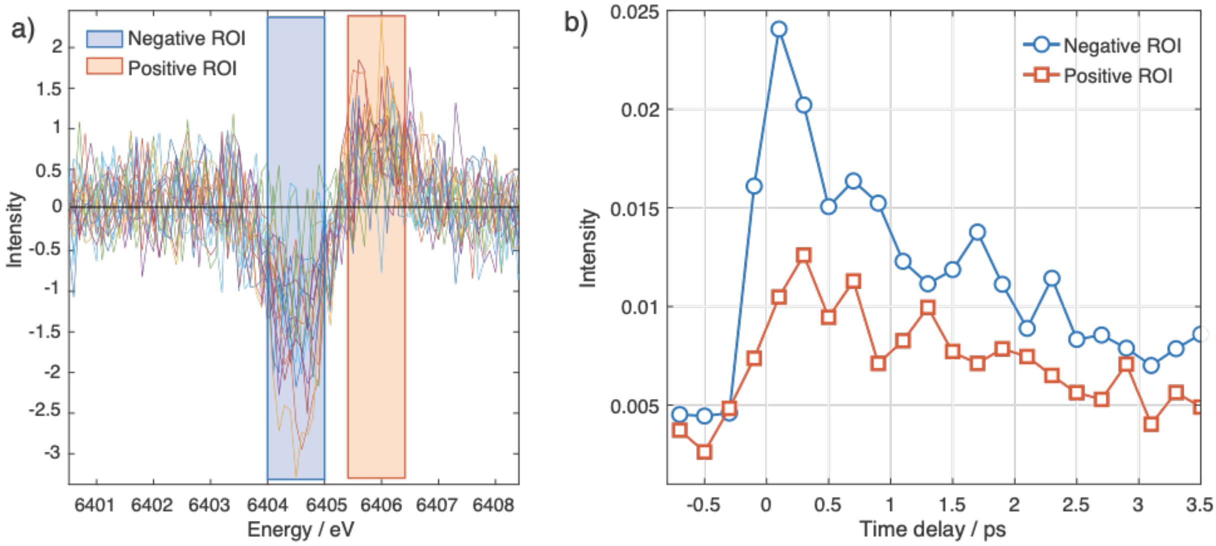

Figure S12: a) Region of interest (ROI) used for integration of the positive (orange box) and negative (blue box) peaks of the transient XES data at various time delays (colored lines). b) Kinetic traces obtained from the integration of the positive (orange squares) and negative (blue circles) ROIs.

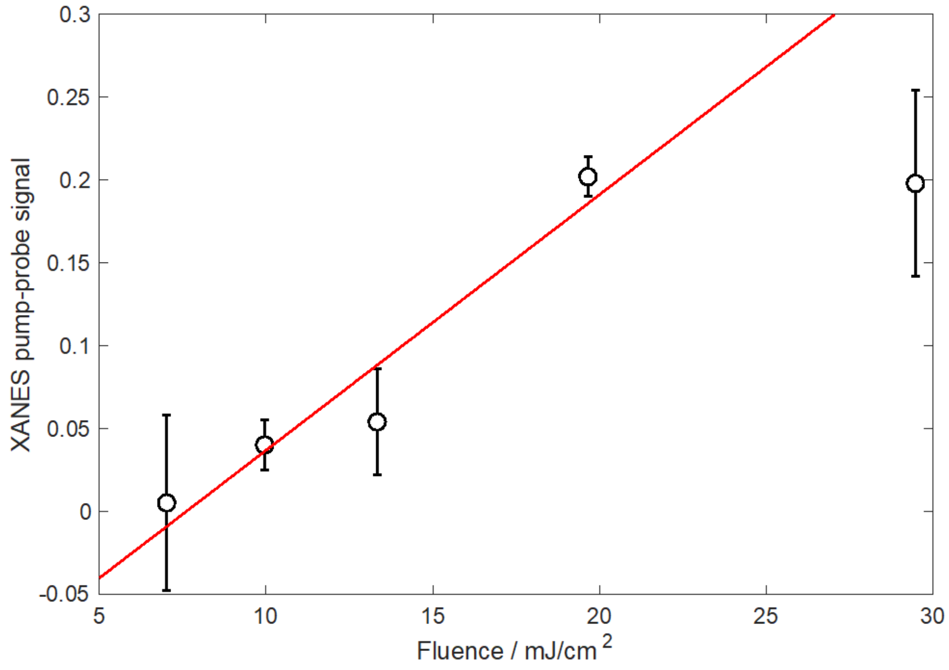

Figure S13: Fluence dependence of the XANES transient signal at 7125 eV and 500 fs time delay after excitation at 350 nm (dots). The dependence is clearly linear up to ~20 mJ/cm^2 (red line).

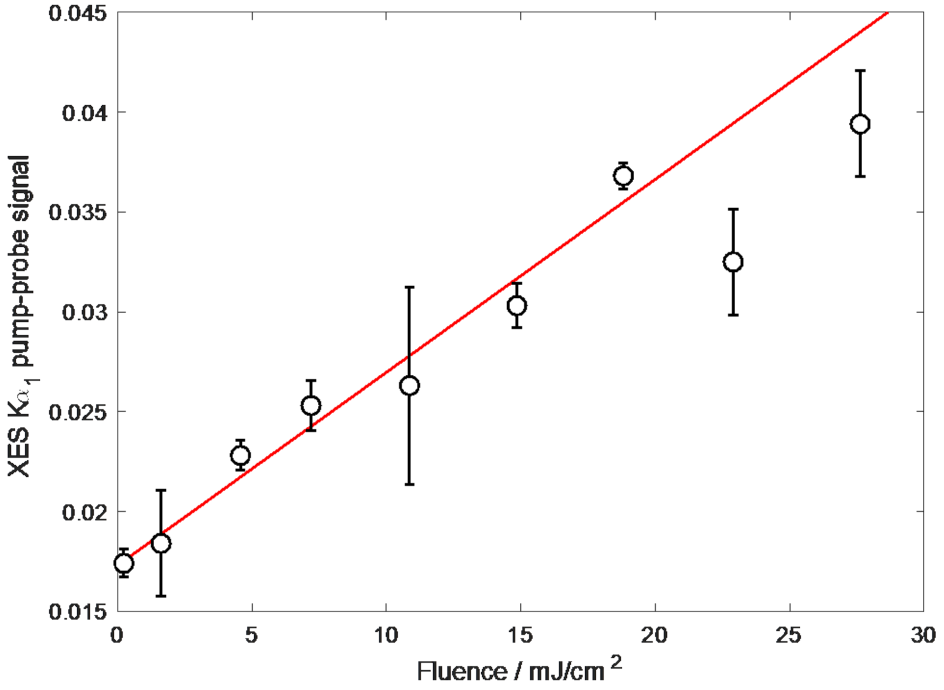

Figure S14: Fluence dependence, recorded at the European XFEL, of the XES transient signal at 6405 eV and 500 fs time delay after excitation at 400 nm (dots). The dependence is clearly linear up to ~20 mJ/cm^2 (red line).

References

1. C. Consani, O. Bram, F. van Mourik, A. Cannizzo, M. Chergui, Energy transfer and relaxation mechanisms in Cytochrome c. *Chem Phys* **396**, 108-115 (2012).
2. M.-C. Cheng, A. M. Rich, R. S. Armstrong, P. J. Ellis, P. A. Lay, Determination of iron− ligand bond lengths in ferric and ferrous horse heart cytochrome c using multiple-scattering analyses of XAFS data. *Inorg Chem* **38**, 5703-5708 (1999).
3. C. J. Milne *et al.*, SwissFEL: The Swiss X-ray Free Electron Laser. *Applied Sciences* **7**, 720 (2017).
4. A. Galler *et al.*, Scientific instrument Femtosecond X-ray Experiments (FXE): instrumentation and baseline experimental capabilities. *Journal of synchrotron radiation* **26** (2019).
5. M. Silatani *et al.*, NO binding kinetics in myoglobin investigated by picosecond Fe K-edge absorption spectroscopy. *Proceedings of the National Academy of Sciences* **112**, 12922-12927 (2015).
6. M. Levantino *et al.*, Observing heme doming in myoglobin with femtosecond X-ray absorption spectroscopya). *Structural Dynamics* **2**, 041713 (2015).
7. M. W. Mara *et al.*, Metalloprotein entatic control of ligand-metal bonds quantified by ultrafast x-ray spectroscopy. *Science* **356**, 1276-1280 (2017).
8. J. M. Glownia *et al.*, Pump–probe experimental methodology at the Linac Coherent Light Source. *Journal of synchrotron radiation* **26** (2019).
9. J. Szlachetko *et al.*, A von Hamos x-ray spectrometer based on a segmented-type diffraction crystal for single-shot x-ray emission spectroscopy and time-resolved resonant inelastic x-ray scattering studies. *Review of Scientific Instruments* **83**, 103105 (2012).
10. G. Palmer *et al.*, Pump–probe laser system at the FXE and SPB/SFX instruments of the European X-ray Free-Electron Laser Facility. *Journal of synchrotron radiation* **26**, 328-332 (2019).
11. M. Schmid, H. P. Steinrück, J. M. Gottfried, A new asymmetric Pseudo-Voigt function for more efficient fitting of XPS lines. *Surf Interface Anal* **46**, 505-511 (2014).
12. C. Consani, G. Aubock, O. Bram, F. van Mourik, M. Chergui, A cascade through spin states in the ultrafast haem relaxation of met-myoglobin. *J Chem Phys* **140** (2014).
13. J. Helbing *et al.*, Time-resolved visible and infrared study of the cyano complexes of myoglobin and of hemoglobin I from Lucina pectinata. *Biophysical Journal* **87**, 1881-1891 (2004).
14. J. Helbing, Spin state transitions upon visible and infrared excitation of ferric MbN3. *Chem Phys* **396**, 17-22 (2012).
15. M. D. Liptak, X. Wen, K. L. Bren, NMR and DFT investigation of heme ruffling: Functional implications for cytochrome c. *Journal of the American Chemical Society* **132**, 9753-9763 (2010).
16. N. Schuth *et al.*, Effective intermediate-spin iron in O2-transporting heme proteins. *Proceedings of the National Academy of Sciences* **114**, 8556-8561 (2017).
17. W. K. Zhang *et al.*, Tracking excited-state charge and spin dynamics in iron coordination complexes. *Nature* **509**, 345-+ (2014).
18. F. A. Lima *et al.*, A high-repetition rate scheme for synchrotron-based picosecond laser pump/x-ray probe experiments on chemical and biological systems in solution. *Review of Scientific Instruments* **82** (2011).
